# Affective-touch experiences and interpersonal space in anorexia nervosa

**DOI:** 10.1007/s40519-026-01886-w

**Published:** 2026-06-23

**Authors:** Federica Toppino, Benedetta Salis, Eugenio Scaliti, Francesco Bevione, Matteo Panero, Annalisa Brustolin, Giovanni Abbate-Daga, Paola Longo

**Affiliations:** 1 Eating Disorders Center for Treatment and Research, Department of Neuroscience, University of Turin, Via Cherasco 11, 10126 Turin, Italy; 2 Human Science and Technology, University of Turin, Vicolo Cesare Benevello 3a, 10124 Turin, Italy; 3 Department of Management “Valter Cantino”, University of Turin, Corso Unione Sovietica 218 bis, 10134 Turin, Italy

**Keywords:** Anorexia nervosa, Affective touch, Body image, Interpersonal space, Bodily self-consciousness, Interpersonal avoidance

## Abstract

**Objectives:**

The present study explored affective touch in anorexia nervosa (AN) by: (a) comparing patients with AN and healthy control subjects (HCs) on the quality and quantity of affective-touch experiences across the lifespan; (b) investigating the association between affective touch and body-image-related symptoms; (c) assessing the link between affective touch and interpersonal space (IPS).

**Methods:**

Participants (76 patients with AN and 77 HCs) completed self-report questionnaires measuring eating-related symptoms, anxiety, depression, and affective-touch experiences. IPS was assessed with a computer-based stop-distance task with different social conditions.

**Results:**

Patients with AN had higher general (i.e., anxiety and depression), and body-image-related (e.g., body checking, body dissatisfaction) symptoms than HCs; moreover, they reported lower quantity of affective touch both in childhood and adulthood and less comfort with affective touch, with a medium to large effect size; more frequent negative affective-touch experiences were also observed in patients than in HCs, with a medium effect size. In patients, lower experienced affective touch, both in childhood and adulthood, and reduced touch-related comfort were associated with larger IPS, independent of anxiety and depression, and with a high effect size after statistical adjustment. No associations with IPS emerged in HCs.

**Conclusions:**

The results highlighted a link between negative and reduced affective touch and social difficulties and avoidance in AN. The study remarks the relevance of affective-touch experiences and their relation with implicit body-related mechanisms, suggesting addressing early tactile experiences and interpersonal functioning in clinical interventions.

**Level of evidence:**

Level III, case–control study based on self-report questionnaires and a behavioral task.

**Supplementary Information:**

The online version contains supplementary material available at https://doi.org/10.1007/s40519-026-01886-w.

## Introduction

Anorexia Nervosa (AN) pervasively affects corporeal experience at the perceptual, cognitive, and emotional levels [9]. Patients with AN, indeed, report a wide range of body-related symptoms, including body dissatisfaction, body-image distortion [28], body-image avoidance, and body checking [29]. While these symptoms are recognizable and explicitly reportable by individuals, they arise from an entangled connection with altered implicit body-related processes, such as body schema, multisensory integration, and interoception [10, 13, 22]. In this context, it was proposed to move from the mere concept of body image to the more complex and exhaustive framework of embodiment, namely, the perception and experience of one’s own body, that influences identity and gives rise to several symptoms in eating disorders [27]. Relatedly, recent researches started demonstrating a deep bodily impairment in patients with AN, including alterations in several implicit body-related functions [16, 22]. Among implicit mechanisms, touch perception is particularly relevant, though it has received comparatively little attention. Previous studies have shown that patients with AN overestimate the distance between tactile stimuli on their arms and abdomen [14] and perceive horizontal tactile stimuli as wider than vertical ones (Spitoni et al., 2015). These data highlighted that distortions in body representation and concerns over body size can influence the processing of tactile information. However, the connection between body-image disturbances and touch perception has not been explored regarding the affective component of touch.

Interpersonal affective touch plays a fundamental role in emotional communication and social behaviors, and is also connected to psychological health, stress management, and body representation [2]. Physiologically, affective touch is mediated by C-tactile afferent fibers, which transmit signals to the dorsal horn of the spinal cord, and from there to thalamic medial and intralaminar nuclei [20, 21]. The signals are then conveyed to several cortical regions, including the superior temporal sulcus, the amygdala, the insula, and the anterior cingulate cortex, which are involved in the emotional evaluation of touch [21].

Alterations in affective touch have been observed in AN. For instance, Crucianelli et al. [6] reported that patients with AN perceived the gentle touch as less pleasant than healthy control subjects, while Davidovic et al. [8] identified altered activity in the dorsal striatum, potentially impacting the evaluation of pleasant tactile stimuli [8].

Despite these findings, no studies have yet explored how the individual history of affective touch relates to both self-report body-image symptoms and implicit body mechanisms involved in social interactions, such as the regulation of interpersonal space (IPS; i.e., the space around the body in which we interact with others). In this context, recent studies have demonstrated that patients with AN prefer a larger distance to interact with others, compared to healthy control subjects [16, 23]. However, the connection between IPS abnormalities and other potentially relevant variables (e.g., early experiences, body-image symptoms) has not been deepened yet.

Since affective touch represents the direct and primary channel of social communication, we suppose that its alteration in AN may influence interpersonal behavior and the modulation of social distance, consistent with the cited IPS disturbance in this population [16, 23].

Given the emerging gaps in the literature, we became interested in exploring affective-touch experiences across the lifespan and their potential link to how interpersonal space is managed in AN. We, therefore, developed the following specific aims: (a) to compare patients with AN and healthy control subjects on the quantity and quality of affective-touch experience during the lifespan; (b) to confirm data on IPS modulation alteration in AN; (c) to investigate the connection between affective touch and explicit body-image-related symptoms, such as body dissatisfaction, body avoidance, and body checking, as well as between affective touch and IPS.

Based on the previously cited literature, we expect to observe fewer and more negative experiences with affective touch, especially during childhood, in patients with AN than in HCs; moreover, we hypothesize that a negative history of affective touch can have an association with the way patients physically interact with others.

## Methods

### Participants

We recruited 76 patients with AN at the Eating Disorder Center of the “Città della Salute e della Scienza” of the University of Turin, Italy. Inclusion criteria were: age above 18 years; diagnosis of AN according to DSM-5. Exclusion criteria were: comorbid psychotic disorder; cognitive or neurological impairment; current use of substances or alcohol.

Participation in the study was proposed only to clinically stable patients, defined as acutely and clinically severe patients who did not present urgent or emerging life-threatening conditions at the time of recruitment. In addition, all patients were receiving psychopharmacological treatment and participated in the study while on a stable pharmacological regimen.

The control sample consisted of 77 healthy individuals recruited from medical students and residents aged 18 years or older. Exclusion criteria included: history of psychiatric disorders; cognitive or neurological impairment; current use of substances or alcohol.

All subjects signed the informed consent. The study was approved by the Ethical Committee of the University of Turin under the registration number CS2/840 (approval date 20/12/2021).

### Materials and procedure

All participants completed the computer-based task to evaluate IPS in different social conditions. The task is a modified version of the stop-distance paradigm, in which participants are asked to choose the preferred social distance from actors (male or female) approaching with different facial expressions (i.e., neutral, gaze turned laterally, friendly, threatening). A detailed description of the task is available in Longo Scaliti, et al. [15] and in the Supplementary Materials (Figure S1).

The subjects completed the following self-report questionnaires:

- Eating Disorder Examination Questionnaire (EDEQ; [4]): to assess the typical symptoms of eating disorders;
- Beck Depression Inventory (BDI; [1]) to investigate depression levels;
- State–Trait Anxiety Inventory (STAI; [26]) to measure current (state) and habitual (trait) levels of anxiety;
- Body Shape Questionnaire (BSQ; [18]), Body Checking Questionnaire (BCQ; [3]), Body-Image Avoidance Questionnaire (BIAQ; [17]) to explore the explicit attitudes toward body and body image.
- Tactile Biography Questionnaire (TBQ; [2]): the 28-item tool assesses the quantity of affective touch experienced during childhood and adulthood, also exploring the emotional valence associated with touch and the presence of negative events related to interpersonal contact.

### Statistical analysis

Statistical analysis was conducted with RStudio and SPSS. Independent-sample *t* test was run to investigate differences in continuous variables between HCs and patients, with effect-size calculation with Cohen’s d; chi-squared was used for categorical variables, with Cramer’s V to assess effect size. The modulation of IPS in the task was analyzed using linear mixed-effects models (LMEM), as described in our previous work [16]. In particular, we ran LMEM with the glmer function from the R package “lme4” to assess the differences in IPS between the two groups across several experimental conditions (please, for further details on the analysis, refer to [16]).

Regression analysis was conducted to investigate the relationship between affective touch and body-image-related symptoms, as well as between affective touch and IPS. In case of significant associations, hierarchical regressions were run to control for the role of depression and anxiety entered as covariates.

Because no formal a priori power analysis was conducted, we performed a post hoc sensitivity analysis with the pwr package in R, in order to estimate the minimum detectable effect size with the current sample size, α = 0.05, and 80% power.

## Results

### Differences between patients with AN and HCs

Patients with AN scored significantly higher than HCs in eating-related symptoms, anxiety, depression, and body-related symptoms (Supplementary Material S1).

**Fig. 1:**
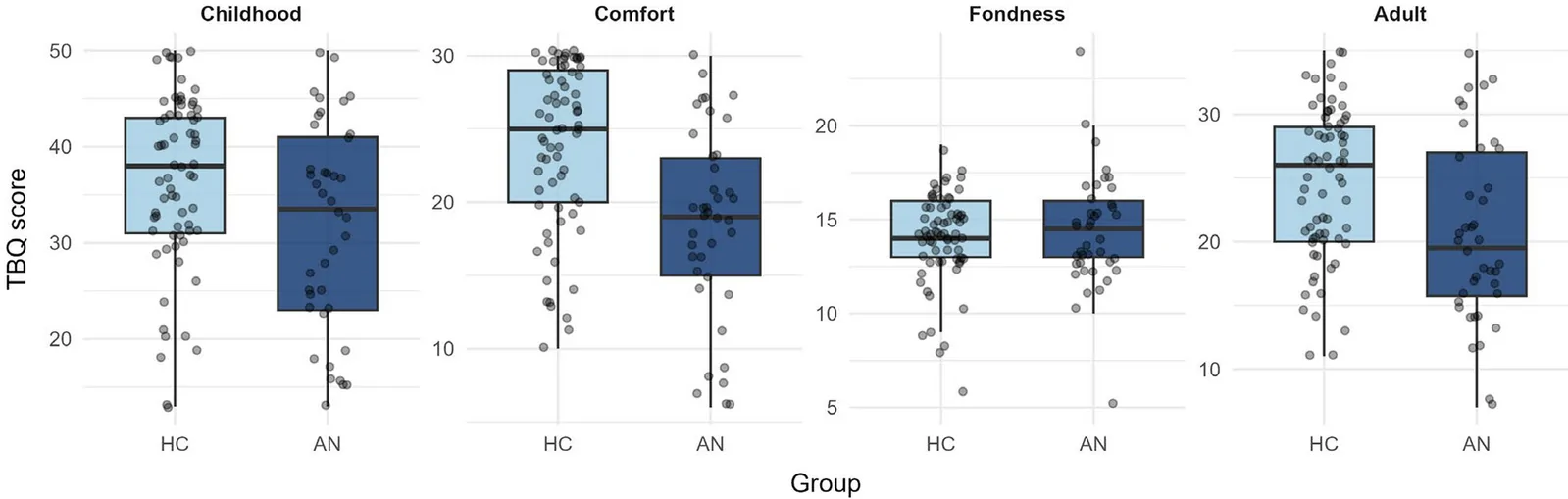
Differences between patients with AN and HCs in affective touch

As regards affective touch, patients had lower scores in the childhood, comfort, and adulthood subscales of TBQ (Table 1; Fig. 1), and reported a significantly higher frequency of negative emotions and negative events related to interpersonal tactile touch compared to HCs (Table 2).

**Table 1:** Differences between patients with AN and HCs in affective touch

	Patients with AN (*n* = 76)	HC (*n* = 77)	T	*p*	Cohen’s *d*
	Mean (SD)	Mean (SD)			
TBQ_childhood	31.8 (10.8)	36.6 (9.0)	2.347	**0.022**	0.489
TBQ_comfort	18.6 (6.5)	23.9 (5.7)	4.235	**0.001**	0.872
TBQ_fondness	14.4 (3.1)	14.0 (2.4)	− 0.675	0.502	− 0.144
TBQ_adult	20.6 (7.1)	24.6 (5.9)	2.987	**0.004**	0.622

TBQ, Tactile Biography Questionnaire

Bold values indicate statistically significant p values (p 0.05)

**Table 2:** Differences between patients with AN and HCs in categorical variables of TBQ

	Patients with AN (%)	HC (%)	Chi-squared	*p*	*V*
Positive emotion	55	83.6	10.357	**0.002**	0.311
Negative emotion	45	16.4			
Negative event	62.5	31.3	9.921	**0.002**	0.304
No negative event	37.5	68.7			

Bold values indicate statistically significant p values (p 0.05)

Finally, patients had a significantly larger IPS than HCs in all conditions (chi-squared = 10.653, *p* = 0.001; Supplementary Material S2).

### Affective touch and body image

No significant associations emerged between TBQ subscales and body-related symptoms (i.e., body dissatisfaction measured with BSQ, body-image avoidance measured with BIAQ, body checking measured with BCQ), either in patients with AN or in HCs (Supplementary Material S3).

### Affective touch and IPS

**Fig. 2:**
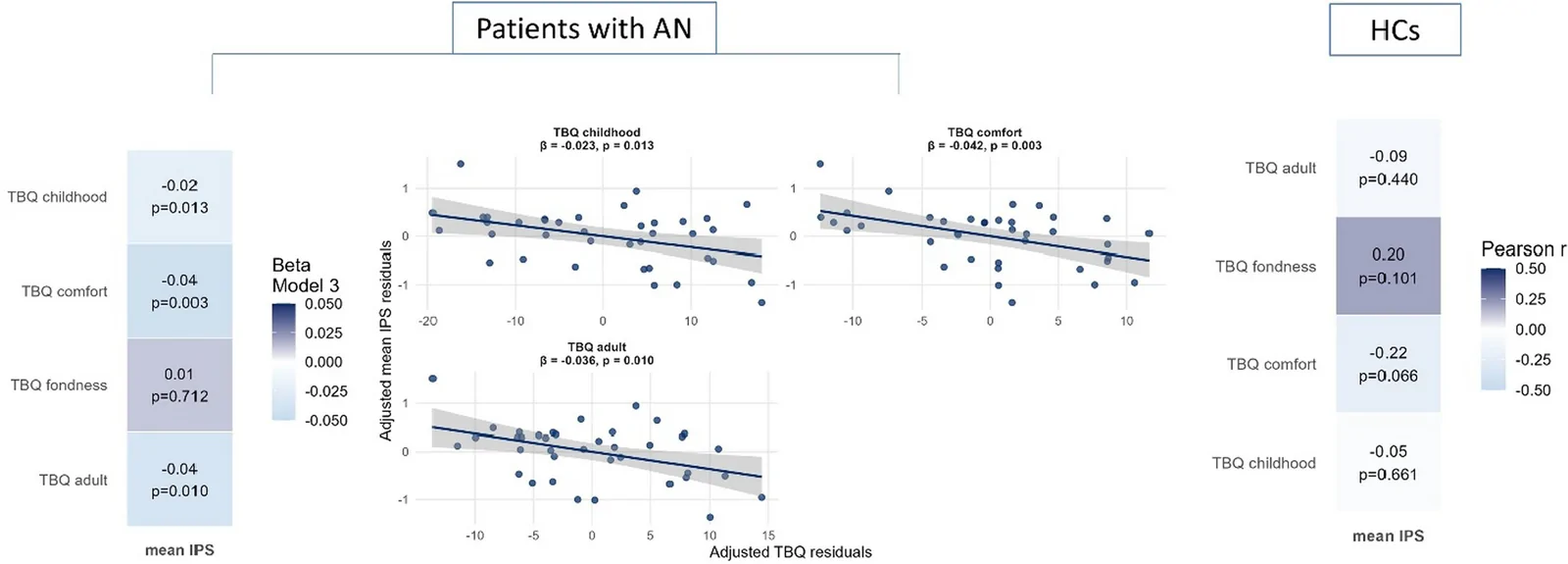
Associations between affective touch and IPS. TBQ, Tactile Biography Questionnaire; IPS, Interpersonal Space. Adjusted associations between TBQ subscales and IPS in patients with AN. Heatmap shows β coefficients from Model 3 (adjusted for BDI and trait anxiety). Scatterplots represent partial regression plots

Negative significant associations were observed between IPS and quantity of affective touch experienced both in childhood and adulthood, and comfort associated with affective touch (Table 3 and Fig. 2); these associations survived the statistical adjustment for depression (step 2) and anxiety (step 3; Table 3).

**Table 3:** Associations between affective touch and IPS modulation in patients with AN

	Step 1			Step 2			Step 3		
	*β*	*R*^2^	*p*	*β*	*R*^2^	*p*	*β*	*R*^2^	*p*
TBQ_Childhood	− 0.019	0.111	**0.035**	− 0.022	0.209	**0.014**	− 0.023	0.217	**0.013**
TBQ_Comfort	− 0.041	0.194	**0.004**	− 0.042	0.270	**0.003**	− 0.042	0.271	**0.003**
TBQ_Fondness	0.022	0.013	0.479	0.013	0.062	0.687	0.012	0.062	0.712
TBQ_Adult	− 0.027	0.101	**0.046**	− 0.036	0.225	**0.009**	− 0.36	0.227	**0.010**

TBQ, Tactile biography questionnaire

Step 1, association between TBQ and IPS; Step 2, adjusted model for depression as measured with BDI; Step 3, adjusted model for anxiety as measured with STAI

Bold values indicate statistically significant p values (p 0.05)

In the group of HCs, no significant associations between affective touch and IPS emerged (Fig. 2).

The post hoc sensitivity analysis showed that the study’s sample size allowed the detection of small to moderate group differences (Cohen’s *d* = 0.46), approximately moderate correlations (*r* = 0.31), and medium to large regression effects (Cohen’s *f*^2^).

## Discussion

The following main findings emerged from the present study: (1) patients with AN, compared to HCs, had higher levels not only in eating-related and general psychopathology, but also in body-related self-reported symptoms; moreover, patients with AN experienced lower quantity of and lower comfort related to affective touch during the lifespan, and reported more frequent negative events associated to affective touch compared to HCs; finally, patients had a larger IPS than HCs; (2) affective touch did not correlate with any of the self-reported body-related symptoms either in patients with AN or in HCs; and (3) in the group of patients with AN, negative associations emerged between IPS and quantity of affective touch experienced both in childhood and adulthood, and comfort related to affective touch; these associations were independent of anxiety and depression.

Before discussing these findings, it is worthy to acknowledge that, given the cross-sectional nature of the study, the results should be interpreted with caution, as no causal conclusions can be drawn from the observed associations.

The lower quantity of affective touch in patients with AN than in HCs could reflect the perception of lack of tactile nurturing and the touch deprivation reported by patients with AN both during childhood and adulthood [11]. Accordingly, in this study, patients had lower levels of comfort in affective touch than HCs, again, in line with the literature [7]. Moreover, patients had a higher frequency of negative events related to affective touch, and this is in accordance with the common presence of physically directed childhood trauma (both abuses and neglect) in patients with AN [15], adding that, beyond the cited traumatic events, other subtle and daily experiences may also occur and contribute to negatively shaping the relationship with the body. These data, indeed, highlight that patients with AN often have a negative history of interpersonal bodily experience since infancy. This may have etiopathogenetic relevance, since it was established that early affective-touch experiences play a crucial role in bodily awareness and self-other boundaries construction [2], which are among the core pathogenetic nuclei of AN. However, future longitudinal studies are needed to confirm the discussed result, since the data could be influenced by recall biases and the acute state of eating-related pathology.

In the present study, patients with AN showed a larger IPS compared to HCs, confirming the previous data on the topic [16, 23], and suggesting a strong disturbance of IPS in AN.

Affective touch did not correlate with any body-related symptoms in either group. This is in contrast to previous studies stating a connection between affective touch and body image [5, 6]. In this context, it is important to acknowledge that this study adopted just self-report measures to assess both body-image-related symptoms and affective-touch history; this could have a role in the disagreement with previous studies and suggests interpreting results with caution due to potential recall biases. Differently, analysis of patients with AN revealed that a lower quantity of affective touch, both in childhood and adulthood, as well as a lower comfort related to affective touch, correlated with a higher preferred interpersonal distance. Crucianelli and colleagues (2016) described that affective touch in patients with AN elicited lower pleasantness than in HCs [6]; relatedly, in the present study, patients with AN reported more frequent negative emotions related to affective touch than the healthy group. These data together led us to speculate that affective touch modulates the IPS: patients can, indeed, connect the interpersonal closeness to an anticipation of affective touch, thus implicitly enlarging IPS boundaries as a defensive mechanism against negative emotions elicited by affective touch; further longitudinal studies are needed to establish the direction of the observed association, and to rule out the potential contribute of eating-related symptoms to the results. Moreover, the observed mechanism might also reflect the broader avoidance tendencies, central in AN [12, 19]. It was indeed described that anorexic symptoms could serve to avoid negative emotions as well as interpersonal relationships [30]; we hypothesized that in this study, the avoidance of interpersonal proximity could aim to avoid, in turn, the negative emotions triggered by affective touch. However, despite the retrospective report on childhood tactile experiences, given the cross-sectional design of the present study, the causal relationship should be interpreted with caution.

It is also worth noting that the association between affective touch and IPS remained significant also after controlling for anxiety and depression. This suggests that the observed relationship is not merely driven by emotional distress; it can be, instead, speculated that there is a specific link between affective touch and IPS, but future studies are needed to confirm this, since our data cannot exclude the possibility that the finding could be a consequence of the eating-related psychopathology.

These results (i.e., affective touch did not correlate with self-reported bodily symptoms, but was associated with IPS modulation in patients with AN) highlighted that low affective touch, especially during childhood, is not linked to the way the body is explicitly perceived, but mainly to the way the body implicitly functions in social interactions with others, leading to larger IPS boundaries. However, longitudinal and mediational studies are needed to further explore the relationship between childhood affective touch and IPS.

Our findings may have clinical implications. In particular, the assessment of patients with AN should include past and current comfort with interpersonal touch. When scarce and/or negative affective-touch experiences are present, patients may benefit from interventions aimed at improving tolerance of safe bodily proximity. Promising approaches include affective-touch-based interventions [24] and virtual reality-based procedures addressing embodiment and body exposure [25]. In particular, given the observed alteration in IPS, virtual reality paradigms can be applied to allow the graded manipulation of interpersonal distance in a controlled context in order to target proxemic regulation. Future studies are needed to test whether these interventions are effective with affective touch and IPS issues.

Overall, the results suggest considering the body-related symptoms of AN beyond the typical symptomatology and reportable body-image disturbances. The personal history of interpersonal contact and affective touch, especially regarding infancy, should also be addressed, particularly in psychotherapy settings.

## Strength and limits

The present study has the main strength of considering multiple facets of bodily experiences, taking into account both the subjective reportable symptoms and the implicit and behavioral perspectives. Moreover, the sample size and the adopted statistical analysis (i.e., mixed methods and covariate correction) contribute to the robustness of the findings.

However, the following limitations should be acknowledged: the use of a cross-sectional design and self-report measures that increase the risk of recall biases, especially for data regarding infancy. Furthermore, a comparison between AN subtypes was not conducted. Finally, given the explorative nature of the study, a power calculation was not conducted; however, we ran a post hoc sensitivity analysis revealing that the study’s sample size allowed us to detect medium to large effects for regressions, and small to moderate effects for between-group comparisons and correlations. Future studies could adopt a longitudinal design and deepen the differences across different eating-related diagnoses beyond AN.

## What is already known on this subject?

There is growing evidence on the complexity of bodily-related disturbance in Anorexia Nervosa, with alterations going beyond the mere body-image symptoms. Relatedly, previous studies described alterations in different implicit bodily functions, including the affective touch and the modulation of the interpersonal space.

## What this study adds?

This study provides new evidence on the complex interconnection among body image, bodily self-consciousness, and interpersonal dynamics, showing that reduced and negative affective-touch experiences are specifically associated with the modulation of interpersonal space in patients with AN. Moreover, new data emerged showing that affective touch was not related to self-reported body-image symptoms; this could suggest a dissociation between explicit body-image disturbances and implicit bodily mechanisms involved in social interactions.

## Supplementary Information

Below is the link to the electronic supplementary material.

Supplementary file 1.

## Data Availability

The data that support the findings of the study are available from the corresponding author upon reasonable request.
