# Supplementary material for: Affective-touch experiences and interpersonal space in anorexia nervosa
Source: Eat Weight Disord. 2026 Jun 23;31(1):78. doi: 10.1007/s40519-026-01886-w (PMC13541973; doi:10.1007/s40519-026-01886-w)
Supplement: Supplementary file 1 [file 40519_2026_1886_MOESM1_ESM.docx]

Supplementary TBQ and IPS

S1. Differences between patients with AN and HCs

|  | Patients with AN (n = 76) | HC  (n = 77) |  |  |  |
| --- | --- | --- | --- | --- | --- |
|  | Mean (SD) | Mean (SD) | t | p | Cohen’s d |
| Age | 25.5 (8.1) | 25.7 (2.5) | .225 | .822 | .038 |
| BMI | 16.3 (3.5) | 21.3 (2.7) | 9.353 | **<.001** | 1.586 |
| EDEQ-restrain | 4.2 (1.7) | 0.6 (0.9) | 15.523 | **<.001** | -2.619 |
| EDEQ_food | 3.6 (1.4) | 0.5 (0.8) | 15.818 | **<.001** | -2.670 |
| EDEQ_shape | 4.9 (1.3) | 1.7 (1.5) | 13.562 | **<.001** | -2.327 |
| EDEQ_weight | 4.5 (1.5) | 1.2 (1.2) | 14.033 | **<.001** | -2.390 |
| EDEQ_tot | 4.3 (1.3) | 1.0 (0.9) | 16.535 | **<.001** | -2.807 |
| BDI | 20.7 (6.8) | 4.7 (4.4.) | 16.164 | **<.001** | 2.780 |
| STAI_state | 61.2 (9.8) | 42.8 (11.3) | 10.029 | **<.001** | -1.745 |
| STAI_trait | 63 (6.8) | 45.3 (8.3) | 13.309 | **<.001** | -2.757 |
| BSQ | 141.9 (41.9) | 65.1 (28.1) | 12.258 | **<.001** | -2.161 |
| BCQ | 72.2 (25.8) | 39.5 (12.3) | -9.091 | **<.001** | -1.644 |
| BIAQ | 49.3 (18.3) | 19.1 (7.6) | 12.145 | **<.001** | -2.183 |

BMI = Body Mass Index; EDEQ = Eating Disorder Examination Questionnaire; BDI = Beck Depression Inventory; STAI = State-Trait Anxiety Inventory; BSQ = Body Shape Questionnaire; BCQ = Body Checking Questionnaire; BIAQ = Body Image Avoidance Questionnaire.

S2. Differences between patients with AN and HCs in IPS task

S3. Associations between affective touch and body image symptoms in patients with AN and HCs.

TBQ = Tactile Biography Questionnaire; BAQ = Body Image Avoidance Questionnaire; BCQ = Body Checking Questionnaire; BSQ = Body Shape Questionnaire.
